# A Systematic View on the Use of History for Current Debates in Sociology, and on the Potential and Problems of a Historical Epistemology of Sociology

**DOI:** 10.1007/s12108-018-9385-1

**Published:** 2018-07-10

**Authors:** Christian Dayé

**Affiliations:** Department of Sociology, Alpen-Adria-Universität Klagenfurt, Universitätsstrasse 65-67, 9020 Klagenfurt, Austria

**Keywords:** Gaston Bachelard, History of the social sciences, Historiography, Sociological theory

## Abstract

For various reasons, among them changes in the global higher education regime and competitive knowledge claims from other disciplines, the field of the history of sociology (HoS) has experienced an increased pressure to justify its own existence during the last decade. Positing that the best approach to justify the existence of a thing is to show its usefulness, the article discusses four types of claims to usefulness made by historians of sociology. The history of sociology can be said to be relevant in (I) shaping and maintaining the discipline’s identity; (II) in providing a rich fund of teaching future sociologists; (III) in informing current research and theorizing; and (IV) in reflecting more broadly on the cultural status of sociology in modern societies. The article then assesses the potential and problems of aspiring a historical epistemology of sociology, a proposal made recently especially in German and Anglophone contexts to link the history of science with its philosophy in the sense described as type III. It concludes that selected principles or ideas of historical epistemology can be very fruitfully applied in HoS. However, the project of transferring the whole program of historical epistemology into HoS is bound to fail. Nonetheless, there is plenty of reason to continue conceiving of HoS as an integral part of sociology.

## A Field in Search of Justification: The Current Status of the History of Sociology

Throughout the last decade, scholars concerned with the history of sociology have experienced an increasing external demand – sometimes tacit, sometimes explicit – to provide justifications for the existence of their field. This demand originated in various places and depending on the place, it was informed by varying interests, some political, some economic, and some intellectual. One of the places where the demand for justification arose for the history of sociology (HoS) was the competition for resources within the discipline.^1^ The fate of the archival records of the American Sociological Association (ASA) is a case in point.

Since the early 1990s, a group of sociologists had discussed the idea to initiate a collection of materials relevant in the history of the discipline.^2^ Quite obviously, one important holding in this regard were the papers of the ASA. Alan Sica, who had just been joining Pennsylvania State University, established the contact between the librarians of his university and ASA. ASA officials were very reluctant, though. Amongst other kinds, the papers comprised both internal memos (including also the minutes of the ASA Committee on Professional Ethics, which was involved in a series of tenure cases) and the correspondence files of ASA’s official journals. Especially with regard to these holdings, it was feared that an archive providing unrestricted access to these materials could be interpreted as a violation of the principle of anonymity that was granted to the proponents. However, a contract was signed between the ASA and Penn State in 1997, stating that all in-house memoranda and reports as well as the complete correspondence files of ASA’s journals were to be sent to the university’s libraries. Over the years, the holdings at Penn State grew to an impressive archive of 588 boxes. However, in the mid-2000s, Penn State hired a new library director who found out that the initial contract between ASA and Penn State had established that the journal-related materials could never be accessed. Balancing scholarly value and space restrictions, Penn State informed the ASA that they would return the materials if ASA kept its strict position on the inaccessibility of the materials. ASA did not agree to change its position, so Penn State decided to not renew the agreement with ASA. This triggered an interesting, but ultimately unsolvable debate about what to do with these 580+ boxes. It centered on the problem of comparing the costs of material storage respectively of professional digitalization with the intrinsic intellectual value of the materials.^3^ In the course of this debate, various scholars submitted statements to the ASA with the intent to emphasize the value of historical self-reflection for the discipline. Hence, a number of statements sought to underscore that the history of sociology must be seen as inherent part of the discipline itself.^4^

The fate of these materials has been one trigger amongst others of a debate that has apparently followed sociology over the last half century. Arguments resembling those made in the Save-Our-Archival-Records campaign were made in a two-part piece by Richard Swedberg, who described HoS as providing the discipline’s “working memory” (Swedberg 2012, 2013). One can also find them in a statement written by Martin Bulmer in 2016, then chair of the History of Sociology section, when the ASA Council on Sections summoned the section to declare its purpose, membership strategies, and justification for existence.^5^ A similar statement had to be produced by the section leaders two years later under the aegis of David Swartz. Most of the arguments put forth on these occasions were not new; many can be found in earlier debates, for instance in what came to be called the “Historicist Controversy” in the late 1970s, or in a series on “World Sociology” run by the *Swiss Journal of Sociology* in the late 1990s.^6^

Further challenges to reflect on the position of HoS and on its justification as a subfield of sociology emerged from shifts in the disciplinary tectonics characterizing the field. The history of the social sciences, and specifically their role in the Cold War era, has climbed onto higher ranks in the research agendas of professional historians over the last years.^7^ This increased interest, in turn, has led to a questioning of whether HoS is a legitimate part of sociology, or whether it would fit better within the disciplinary bounds of history. Far from building a consolidated field of research, current studies on the history of sociology do not converge around a gravitational point – neither theoretically, nor methodologically, nor organizationally, in journals and associations.

In parallel to these developments, sociologists researching the history of their discipline have also experienced an urge to explain the usefulness of their research to university administrators. In an attempt to create a more transparent distribution system of reward and resources, universities have become oriented towards impact factors and other alleged indicators of academic relevance. The proliferation of new governance regimes and, especially in Europe, the related structural and intellectual remodeling of teaching in the universities obviously caught historians of sociology off guard. However the objective side of this developments might appear, they subjectively experienced a loss of terrain.

And yet this felt external pressure to self-reflection also had its positive consequences. From a methodological point of view, the question why one should write the history of sociology is inseparably related to the question how one should write it. Thus, the enforced debate on the value and future of the field built the ground for a renewed interest in its methodological foundations, principles, and potentials. Several publications acknowledge this renewed interest. In 2009, the Italian journal *Sociologica* featured an essay by Jean-Michel Chapoulie, who proposed a framework for the history of social and behavioral sciences (Chapoulie 2009a) which was followed by comments by Daniel Geary (2009); Johan Heilbron (2009); Jennifer Platt (2009); Sica (2009); see also the rejoinder by Chapoulie 2009b). Comparing the historiographies of various social science disciplines, the contributions to a volume edited by Backhouse and Fontaine 2014 addressed the questions how, by whom, and to what effect these histories had been written. The 2nd edition of the *International Encyclopedia of the Social and Behavioral Sciences* included an entry on methodological and historiographical approaches and rationales (C. Fleck and Dayé 2015). Also, a collection of essays edited in German by Dayé and Moebius (2015) explicitly addressed the questions how and why one should write the history of sociology, as did a recent article by George Steinmetz (2018).

Assuming that one of the most fruitful roads to secure a place for the history of sociology within sociology is to underscore its (potential) use for the current practice of sociology, the present article proposes a schematic systematization of the varieties of answers given in these recent publications – as well as in the debates referred to above – to the questions how and why one should write sociology’s history. Certainly, taking this assumption as a starting point is not unproblematic. Why should HoS strive for being useful? Isn’t usefulness too restricting a principle to allow for “good” historiography? And indeed, in the literature reviewed, several authors argue that they prefer to do history of sociology for its own sake. Answering to the question why reading the classics, Gianfranco Poggi once claimed that one should do so just because they are useless (Poggi 1996; Poggi is in part reacting to Alexander 1996). Apparently, it wasn’t historians of sociology who made usefulness a standard in discussing the field’s value. As the above suggests, this category entered the discourse from outside the field, or, perhaps more precisely, appears to have been a reaction toward the felt pressures from outside the field. Associations for the advancement of “useful” knowledge – among them the Akademie gemeinnütziger Wissenschaften founded 1754 in Erfurt, Germany, and the American Philosophical Society for the Promotion of Useful Knowledge founded 1766 in Philadelphia (cf. Burke 2016, p. 98) – existed long before there was HoS in any true sense. Yet, discourses aren’t under anyone’s complete control, and once an idea has entered a discourse, it is much harder to make it disappear again. Because of the intrinsic link between the questions of how and why to write the history of sociology, the scheme I present on the following pages is as good a means of HoS’s discourse politics as it is a contribution to its process of methodological self-reflection.

In principle, two answers are possible to the question of whether the history of sociology has anything to contribute to current problems in the discipline: yes, and no. While proponents of the no-side sometimes put forth innovative arguments to corroborate their skepticism, in what follows I focus exclusively on the yes-side. It should be kept in mind that I am not so much concerned with the question whether or not research in the history of sociology actually contributes to current debates; rather, I theorize *how* this *could* be done. By and large, there are four types of positive answers. One can argue that the history of sociology is important (I) because it offers a source for collective conscience and *disciplinary identity*; (II) because it provides and maintains an important way to *teach* new generations of scholars; (III) because it can *inform* current sociological research and theorizing; and (IV) because its allows to *reflect* on the shape as well as the broader cultural and social impact of sociology.

The paper proceeds as follows: the following section discusses these four types in more detail. One of the remarkable features in the philosophy of science is that the question why one should do research on a specific phenomenon is inseparably linked to the question how one should do so, and vice versa. Therefore, the discussion also has to investigate which research approaches seem appropriate for each of the four types of claims. In order to do this, I will refer to recent studies I deem exemplary. The adjacent section focuses on the third type of answers, the claim that history of sociology can (and should) inform current research and theorizing. Especially in the German speaking countries, this claim has recently been repeatedly made under the label “historical epistemology.” At first, the claim concerned mainly the natural sciences (Daston 1994, 1997; Renn 1995; Rheinberger 1997, 2010a, b), but it was later extended to include the social sciences and humanities as well.^8^ As critics have argued, the use of this label owes more to positioning strategies than to historical or theoretical veracity. The programs of Daston and Renn, as Cristina Chimisso (2003, p. 298) once put it, “share the name but not the methods and aims” with those doctrines that have been at the center of debates in those academic circles where historical epistemology originally emerged: in France.^9^ True, historical epistemology has never been a homogeneous field, and the spectrum of positions it encompasses is large. Yet, the recent revival – or, as Yves Gingras (2010) suggests, “branding” – of historical epistemology is highly selective with regard to its main sources, thereby neglecting the continued influence of certain authors on intellectual debates especially in France.

Chief among those original figures that have been neglected by the recent programmatic statements in historical epistemology is the French philosopher Gaston Bachelard (1884–1962), whose project was to search for deep-seated, cultural imaginations informing scientific thought and to establish a psychoanalysis of the scientific mind. This is not to say that Bachelard’s ideas are not present in contemporary historiography of the (social) sciences. Especially through the conveyance of Michel Foucault and Pierre Bourdieu, who both took up parts of his ideas, some of the tenets of Bachelard’s historical epistemology have been followed in recent contributions to the history of sociology literature. However, this happened quite often without reference to the source of these ideas, and to the system of thought that had originally environed them. Bachelardian historical epistemology, thus, suffered a double marginalization: a first out of strategic interest in claiming positions in the academic attention space, and a second out of unfamiliarity of historians of sociology with the philosophical traditions out of which came authors that are more famous in sociology, like Foucault and Bourdieu.

If this situation justifies a more detailed consideration of Bachelard’s thought, it does not guarantee that such a move of reversion results in an added value for the questions why and how to write the history of sociology. Indeed, as I show below, the attempt to approach the history of the social sciences in a Bachelardian spirit encounters various difficulties. The subsequent conclusion elaborates on what I think should be the consequences of these various uses for HoS as a field and for the discipline as a whole. If one accepts the answers that historians of sociology have given to the question of usefulness (or some, at least), there is ample reason to maintain its affiliation with sociology.

A final caveat might be appropriate. When describing the various approaches, I give a few examples of works that I think fit. It is important to keep in mind that these are examples sought to make the point; I do not provide a full literature review here. Also, it cannot be expected that the authors of these works agree with my interpretation of their work nor with the philosophical implications of their location in the scheme. Some authors might feel their text belonged to another category, or perhaps to several. However, the intended meaning is always only a part of the message, another one being the process of reception. Also, the analytic value of a scheme comes from simplification, and the same principle applies to my use of studies.

## The Uses of the History of Sociology

**Fig. 1:**
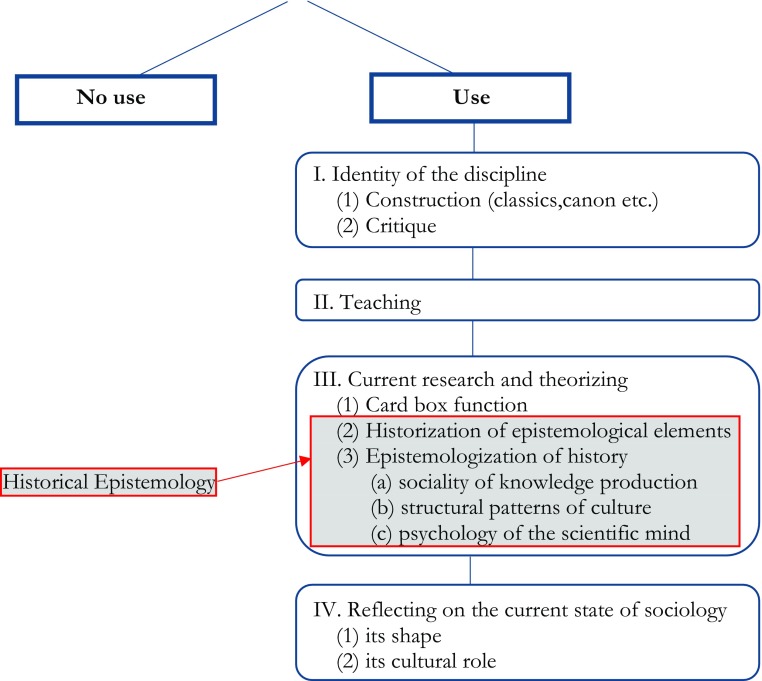
Has the history of sociology any use for the current practice of the discipline, and if yes, which ones?

This section discusses the four types of answers given to the question how HoS can be of use to sociology (see Fig. 1): that it shapes and maintains the identity of the discipline (I); that it offers invaluable teaching materials (II); that it can inform current research and theorizing (III); and finally, that it provides starting points for assessing and reflecting on the current state of the discipline (IV).

### Type I: History as a Means of Identity Politics

The first type of arguments justifying the existence of HoS concerns identity politics. A shared understanding, so the argument goes, of the history of the field is essential to forging the disciplinary identity of sociology, because it provides current scholars with figures, events, and ideas that strengthen, in Durkheimian terms, the collective consciousness of the sociological community and fosters solidarity among its members (I.1). Just like any other social collective, the identity of a scientific discipline is not pre-given, but the complex result of a continuous cultural discourse. Sociology’s identity, thus, can only be fully grasped by conceiving of it as a multidimensional phenomenon. It plays out at various levels, among them a cognitive identity, a social identity, and a historic identity (cf. Lepenies 1981). As a consequence, there are of course differences among the works proposing this argument. Nonetheless, the intent to shape or reflect on the identity of the discipline was the impetus of many contributions to HoS throughout the centuries, which very often took the form of monographs or edited volumes. Prominent examples are Harry Elmer Barnes’ *An Introduction to the History of Sociology* (1948), John Madge’s *The Origins of Scientific Sociology* (1963), Tom Bottomore and Robert Nisbet’s *A History of Sociological Analysis* (1978), but also Alvin W. Gouldner’s *The Coming Crisis of Western Sociology* (1970), Wolf Lepenies’ *Die drei Kulturen* (1985) and Craig Calhoun’s *Sociology in America* (2007).

As especially the last three books mentioned indicate, the identity of the discipline of sociology is a field of knowledge politics, reputation strategies, and power struggles. Lepenies’ book thus had the identity of the field itself as the object of study. With regard to American sociology, for instance, it has been repeatedly argued that, secularist rhetoric aside, the discipline at the deepest levels still adhered to principles of Christian morality (Vidich and Lyman 1985; Smith 2014).

Gouldner’s book as well as some of the chapters of the volume edited by Calhoun in turn challenge the dominant narratives creating a disciplinary identity (I.2). This, of course, is important. The selection of “historic” figures, events, and ideas can and should be criticized and challenged, since it per force reflects the historical and social position of the person who selects. This has been a recurrent motif in many studies that sought to re-establish forgotten or allegedly marginalized scholars, such as Jane Addams (Deegan 1988; Schneiderhan 2015) or W. E. B. DuBois (Morris 2015). Various edited volumes complete the picture (e.g., Goetting 1995; Law and Lybeck 2015). However critical they may be of mechanisms of power and memory within the discipline, these books take a consistently positive and constructive position towards sociology. They want to make it better. By showing the constructedness of the disciplinary identity and opening debates aiming at reformation, such critical reflections on the canon of the discipline have the potential to reinforce the binding forces of the discipline, provided that these reflections are granted space. The construction of a canon and its critique are just two sides of the same coin.

### Type II: The History of Sociology as a Resource in Academic Teaching

The second type of answers emphasizes the didactical value of sociology’s rich past. To learn how earlier luminaries did sociology is presented as one of the most important ways to learn how to do good sociology today. Classics play a major role in this regard, as evinced by works like Lewis Coser’s *Masters of Sociological Thought* (1971), Raymond Aron’s *Les étapes de la pensée sociologique* (1976), originally published in 1967, or Dirk Kaesler’s two volumes *Klassiker der Soziologie* (2003a, b), first published in 1999.

How exactly the classics can be used in teaching the next generations of sociologists is divisive. Looking back from today, the findings of the classics are outdated; their empirical procedures do not meet current standards – some of them did not even meet the standards of their time; most theories put forth by the classics have become refuted since their original formulation; and nobody wants students to emulate their style of writing. What, then, can young and aspiring sociologists learn from reading the classics?

In what remains to be the most systematic treatment of how classic sociological works are used, Arthur L. Stinchcombe (1982) discerned six functions. They can serve as *touchstones*, “examples of beautiful and possible ways of doing scientific work.” (Stinchcombe 1982, p. 2) Second, they offer *developmental tasks*, inviting the student to replace every day clichés about the social world and to engage in the attempt to grasp an argument of high order complexity. The third function of classics is the *small coinage* function. Citing a few classic works in the first pages of your paper serves to inform the reader to which intellectual tradition the paper at hand aspires to belong. Fourth, sometimes current work addresses highly *fundamental ideas*, and many classics offer treatments of these ideas that help to orient one’s own research simply because they paint with a big brush. “In this case we praise the classics for being both unique and fundamental, rather than for being fine work as in the touchstone function, for being complex as in the developmental tasks function, or for being symbols with agreed-on meanings for the small coinage function.” (Stinchcombe 1982, p. 3) Fifth, classics also contain hypotheses which students can decide to test empirically – and thus serve as a starting point for *routine science*.

Finally, and similar to what has been discussed in the preceding section, classics can serve as a resource for a sense of disciplinary solidarity. This is their *ritual function*. Stinchcombe writes:

We define what holds us together as sociologists in part by having a common history. So ritual myths about Max Weber’s staring at a wall in nervous prostration, Georg Simmel being kept from a professorship for being Jewish, Thorstein Veblen refusing the Presidency of the Economics Association because it wasn’t offered when he needed it […], Parsons’s dissertation on some obscure German’s ideas about capitalism, all serve the functions that the cherry tree and the Gettysburg address written on the back of an envelope do in American history. And like the cherry tree and the envelope myths, the fact that I don’t really know whether any of them are true indicates less about the quality of my scholarship than it does about the ritual function of these classics. (Stinchcombe 1982, p. 4).

Leaving aside Stinchcombe’s remarks on the identity shaping function of classics, which has been discussed in some detail in the previous section, we can sum up his answers to the question which use the classics have in teaching by emphasizing that classics offer orientation, both to the newcomers and the long-established dwellers of the sociological village. They provide inspiration for empirical or theoretical projects, and they facilitate mutual understanding by providing quick-to-read clues. Most importantly, classics can be used to teach students what it means to approach things from a sociological perspective (cf. O’Rand 1982; Woodward 1982). Thus, while they might not learn “good” theory from engaging with the classics, students of sociology can learn the basics of good theorizing.

But what, then, would be the task of HoS in this respect? With the all materials available, how could HoS contribute intellectually to the way in which sociology is taught? I give a twofold answer to this. First, for some of sociology’s classics, there exist book production industries eager to publish new biographies on these past scholars. The task of HoS can be to produce works of this and other genres concerning the lives and works of the classic authors, thus ensuring that there is new input to the functions detailed above. Further, and probably more important, there is also the related industry on sociology textbooks. While most of the current introductory textbooks have a section on the history of sociology, and some refer to classic studies in subsequent chapters, I think that this by far does not exhaust the didactic potential of HoS. An introductory textbook to sociology written by historians would look quite different, I presume, than what is currently available. It would perhaps look more like the three classic examples referred to above, which, as I am told, are no longer used in teaching. But it would also include other topics in the history of sociology, like the development and social life of methods, the transformations caused by technical and infrastructural change (the introduction of computers, the founding of international associations, the activities of large funding schemes etc.), or the relation between larger social and cultural processes and the status of sociology as a discipline.

### Type III: History Informing Current Research and Theorizing

Repeating parts of the argument made by proponents of the use of classics in teaching, several authors argue that the history of sociology should be written in a way that renders it useful to current research and theorizing. The claim is that the works of today’s sociologists would become more sophisticated if they were cognizant of, and sensible to, sociology’s past. Beyond sharing this basic conviction, as with type II, there is considerable heterogeneity among the answers subsumed as type III. And as with type II, some of the confusion around this claim might be clarified if the internal heterogeneity is acknowledge and structured.

There appear to be three distinct kinds of answers in type III. Some scholars emphasize what I – inspired by Niklas Luhmann’s fabled “Zettelkasten” – propose to call the *card box function* of history (III.1). Thus conceived, the task of HoS is to establish and maintain a record of important figures, works, ideas, findings, and events, and to be able to provide information upon request. Such a position was implied in Herbert Gans’ (1992) analysis of sociological amnesia, the effect that the discipline appears to have a memory that only rarely extends beyond a timespan of twenty years. Sampling over a variety of fields, Gans found that only 20% of the citations made in the texts he surveyed referred to publications older than this threshold. While he posited that “collective memory can be intellectually and otherwise inhibiting,” he also maintained that “the relative dearth of collective memory in sociology probably also helps to explain the lack of cumulation.” (Gans 1992, p. 708) Studies were repeated not to replicate earlier work, but in complete ignorance of them. And this was an inefficient use of the cognitive potential of sociology as a science.

In a similar vein, Richard Swedberg (2012, 2013) proposed to conceive of HoS as providing the working memory of the practicing sociologist. He argued that some core insights from cognitive science were relevant to reformulating the task of HoS. Among these insights, he singled out that “memory is not so much about the past as it is about the present.” (Swedberg 2012, p. 11) We need our memory in order to navigate our daily lives. It provides a means of orientation without which we would be lost. Further, recent research also shows that human remembering is an interpretive and partially creative process. It is wrong to compare it to the mechanical processes of data storage used in computer technique. Memory is created in the present, and while parts of it are past experiences, new interpretations, contextualizations, links to other memories, and evaluations are added every time we remember. Thus, in addition to the card box, we also need a human brain to use it.

Translating these insights into principles for HoS leads us, as Swedberg maintains, to reconsider how historians of sociology select their topics. If one of the major tasks of HoS is to provide and maintain the discipline’s working memory, then historians of sociology should increasingly work on issues of immediate interest to practicing sociologists. If concepts like status, or valuation, or recognition, or social inequality re-appear at the research front, it might prove worthwhile to research the history of these concepts. If experiments re-appear as the method of choice for many sociologists, it might be good to write their history. In order to enter a conversation that, at worst, reduces the risk of repetition and, at best, increases the level of theoretical or methodological sophistication of current research, historians of sociology should be aware of the trends of the whole discipline and ready to follow them in their own research.

Other writers have similar ideas about how historical research might be useful for actual research and theorizing, but their vision of the task of the historian is different. It moves from memorizing, however active a process, the discipline’s past to a more dialectic partnership between sociology’s past and present. Also, the point of reference is not so much cognitive science, but historical epistemology, a direction in the history and philosophy of science emerging in reflections by scientists like Ernst Mach or Henri Poincaré that came to blossom in the decades later, especially in the works of Ludwik Fleck, Gaston Bachelard, Alexandre Koyré, Thomas Kuhn, Georges Canguilhem, and Michel Foucault. As one can see from this list, the term historical epistemology comprises a very diverse literature. Notwithstanding, Rheinberger (2010b) proposed to identify as the common principles two movements of thought, the *historization of epistemology* and the *epistemologization of history*. While, Rheinberger contends, these two movements are intrinsically interrelated, it is helpful to differentiate them to get a clearer picture of how HoS can be of use to current sociological practice.

With good reasons, historians of sociology claim that it is important to historically contextualize the crucial elements of sociological knowledge production – theories, methods, methodologies, concepts etc. (III.2). The expectation is that by describing the contexts in which specific ideas were formed and identifying how contextual factors influenced the actual shape of cognitive elements, the level of sophistication with which these elements are used in current research and theorizing increases (cf. Reed 2010). This is because a historization results in a more complex picture of the original idea and at the same time identifies those aspects of a cognitive element that were concessions to the historical context and do not convey epistemological value. I select the formulation “complex picture” to avoid the term “nuanced” (cf. Healy 2017), and this points of course to a major pitfall of historization. It can be potentially indefinite. And it can lead to treatments that leave the reader no other choice than to ask “So, what?” Just like in theorizing, bad historization claims to provide a richer or more sophisticated understanding of a piece of knowledge “by adding complexity to it, usually by way of some additional dimension, level, or aspect, but in the absence of any strong means of disciplining or specifying the relationship between the new elements and the existing ones.” (Healy 2017, pp. 118–119) Historical contextualization, as any contextualization, crucially depends on the link between the contextual factor explored and disciplinary knowledge.

Since many elements of knowledge production transgress disciplinary boundaries, most contributions to this literature are to be found outside sociology – just think of the seminal work of Hans Blumenberg (2015, orig. 1987) on the history of theory or the more recent book by Stephen Kern (2008) on the history of causality. An excellent example of how the historization of epistemological elements can play out in HoS is Peter Baehr’s *Hannah Arendt, Totalitarianism, and the Social Sciences* (2010), which, after discussing how Arendt’s understanding of totalitarianism was shaped in a series of interactions with colleagues, uses the thus theoretically enriched concept to understand contemporary radical Islam.

Other scholars embark on projects that speak more to the second movement of thought, the epistemologization of sociology’s history (III.3). They investigate the past with the purpose of identifying those factors that have an impact, both fostering and inhibiting, on the production, dissemination, and evaluation of knowledge (cf. Camic et al. 2011). Thus, their primary interest is not historical, but sociological, or psychological, or philosophical. The history of sociology is taken as a strategic research material to explore, for instance, social processes of recognition, structural patterns of cultural reproduction, or the impact of cultural myths on science.^10^ Scholarly work that is interested in using the history of sociology to elucidate the production falls into three camps. The first camp (III.3.a) emphasizes the sociality of knowledge production, dissemination, and evaluation. Some examples might be appropriate. While writing a history of the Hawthorne experiments, Richard Gillespie’s *Manufacturing Knowledge* (1991) provides rich insights into how the large network of actors and resources that was constructed around these experiments, and the heterogeneous interests brought together in this alliance, shaped both the way the research was designed and the further career of the results it produced. Relying on Randall Collins’ theory of interaction ritual chains and their importance for intellectual life (esp. Collins 1998), Savelsberg and Flood (2011) looked at the field of criminology to explore how intellectual stimulation and emotional energy were channeled through specific network ties. Similarly, in *Becoming Mead*, Daniel R. Huebner (2014) uses “George H. Mead” to explore the social foundation of academic knowledge production. How, Huebner asks, do we know about other scholars? How do scholarly communities create, communicate, accumulate, and forget these understandings? Mead, a man who “is known in a discipline in which he did not teach for a book he did not write” (Huebner 2014, p. 3), appears as a predestined object to answer such questions. And finally, Sarah E. Igo’s *The Averaged American* (2007) asked how the introduction of large surveys and opinion polls in America transformed the understanding of normality, and the sources by which people formed this understanding.

A second, yet sparsely populated camp is interested in using the history of sociology to explore more general patterns of cultural reproduction (III.3.b). The best example here is Andrew Abbott’s *Chaos of Disciplines* (2001), which argues that the mechanism operative in the history of social science – a sequence of fractal distinctions and remapping – can be found in any kind of interacting cultural system.

Even less populated is the third camp when it comes to the history of sociology. Nonetheless, work on other scientific disciplines suggest that this camp has something to offer to HoS. The main idea of this camp is to approach the history of a science with an eye on obstacles that inhibited earlier scientists to see the true nature of things (III.3.c). Scholars in this camp seek to identify cultural and psychological boundaries to the scientific mind, boundaries that make it hard to even *conceive of* a certain idea. An early treatise of this idea is of course Ludwik Fleck’s *Genesis and Development of a Scientific Fact* (1979, orig. 1935), in which he defined thought style as unavoidable frame of reference for any insight. Fleck’s essentially epistemological/psychological argument however was eclipsed in the process of reception, and his more sociological ideas on how thought collectives function as bearers of thought styles were more vividly discussed. The epistemological line of thinking has also been developed very radically in the works of Gaston Bachelard, especially in his books *The New Scientific Spirit* (1984; orig.: Le nouvel esprit scientifique, 1934) and *The Formation of the Scientific Mind* (2002; orig.: La formation de l’esprit scientifique, 1938). In these books, Bachelard sought to establish a psychology, or even psychoanalysis of the scientific mind. In contrast to Fleck (and Kuhn), Bachelard was certain and explicit that there was scientific progress. Based on this conviction, he formulated a normative and deliberately presentist program. He argued that, in order to be of use, the history of science had to evaluate earlier science by current standards. Only such a normative approach would allow to identify obstacles that hindered the progress of scientific rationality.

### Type IV: Reflecting Sociology’s Current State

Finally, a fourth type of answers to the question of the use of the history of sociology refers to the value of historical research to reflect on the current state of the discipline. These reflections can emphasize two levels. Firstly, historical data on the development of sociology is useful in understanding its current shape (IV.1). The classic, and in a sense paradigmatic study in this regard is Stephen Turner and Jonathan Turner’s *The Impossible Science* (1990). Focusing on the material, symbolic, and organizational resources available to American sociologists since the final decades of the nineteenth century, they trace the failures of the repeated attempts to turn sociology into a “science.” While not necessarily following their theoretical argument, the focus of Turner and Turner on resources has been used in several other studies.^11^ The frame of reference for this type of studies is mostly national, which is justified since the policy systems in which disciplines operate are still largely national. Such work allows for assessing the historical processes that gave the discipline its current shape. This helps to consider new paths of development, especially when compared to other national histories of this type. This was the explicit aim of some recent research projects in this area which established historical datasets that allow for comparison both across countries and across social scientific disciplines.^12^

Another level where the history of sociology is used to reflect on its current state emphasizes the broader social and cultural role of sociology (IV.2). Modern societies entrust large parts of their self-observation to the social and cultural sciences. These sciences, and sociology chief among them, are thus themselves producers of culture, and should as such be subjected to a reflexive analysis informed by (classic) sociology of knowledge (cf. Endreß 2015; Srubar 2015). If sociology takes itself seriously as a discipline concerned with social and cultural forces and processes, it must *per force* explore its own role as such a force within modern society. It must explore the discursive power relations that structure the space of sociology in society, and it must investigate whether, or to which extent, its leading ideas are socially determined by the cultural, economic, and political positions of its members. Conceived as a historical sociology of knowledge about the social, HoS would function as a corrective to the leading discourses within the discipline.

## Gaston Bachelard and the Normative Historiography of Science

Many recent contributions to the HoS literature, however, have shown a different corrective claim than the one just mentioned. Instead of reflecting on the discipline’s position within society, they focused more on the intellectual content and attempted to improve our understanding of sociological theorizing and research – past *and* present. As I argued above, this approach of linking the history of science with its philosophy has been called historical epistemology in the German (and Anglophone) countries, which introduced confusion especially among scholars cognizant of French intellectual history. This recent “revival” has covered up the roots of historical epistemology in the works of Gaston Bachelard, Georges Canguilhem and others.^13^ In HoS, their ideas were partly present, yet not in their original form, but in the form they received in the works of Michel Foucault and Pierre Bourdieu, who have received far more attention within sociology than their forefathers. This article will thus, in a first step, discuss how Bachelard conceived of the relation between history of science and philosophy of science, and then review which of his ideas were taken up by his more prominent pupils.

To start with a superficial similarity: Both Bachelard/Canguilhem and Foucault/Bourdieu have a more or less explicit interest in a normative historiography.^14^ At the time when Gaston Bachelard came to the Sorbonne in 1940 as a professor of history and philosophy of science, a metaphor very common in French philosophical circles to describe the relation between the history and the philosophy of science was the laboratory. It was the place where the theoretical ideas formulated by the philosophers could be tested against reality (cf. Chimisso 2003, p. 305; Brenner 2006). While this metaphor relies on the supposedly objective practice of controlled observation, Bachelard, as well as his successor as the director of the Institut d’histoire des sciences, Georges Canguilhem, proposed a different role for the history of science. Its task was normative. It was to evaluate and judge past forms of science. This program of a normative historiography of science was initiated by Bachelard and completed in the works of Canguilhem.^15^ While it is possible to differentiate between the contributions, convergences and divergences of these two scholars, this is primarily of philological interest and will not be done here (cf. Chimisso 2013; Roudinesco 2008). Instead, the focus of the ensuing narrative is on Bachelard, but this Bachelard is a persona composed of both his own writings and the presentation of his thoughts in the writings by Canguilhem.

Bachelard argued that in the face of the rapid progress of the sciences, philosophy of science had to give up its a priori and normative stance towards science and to begin to reflect *ex post* on scientific practices. In his view, philosophy of science had to become an *ancilla scientiae*. It was not able to dictate ex cathedra how science should be conducted properly. Rather, philosophy of science had to adapt its terminology and structure in order to comprehend the flexibility and rapid evolvement of contemporary science (Bachelard 1984, p. 10, 1974, pp. 16–29).^16^ Writing a normative history of science meant that the analysis provided insights which could be used to re-consider, re-orient, and calibrate the fundamental epistemology. The term epistemology, however, was understood broadly. It did not refer to a systematic theory of knowledge, but instead to an active way of reflecting “on the historical conditions under which, and the means with which, things are made into objects of knowledge.” (Rheinberger 2010b, p. 2) In contrast to the Anglophonic (but also the German) tradition, such a conceptualization of epistemology is also interested in scientific practices and, more generally, the context of discovery.

The metaphor Bachelard used to describe the relation between philosophy of science and the history of science was not the laboratory, as some of his contemporaries did, but the courtroom, or tribunal (Canguilhem 1994a; Chimisso 2003). This tribunal had to assess and pass judgment on past scientific ideas. However, this metaphor required an important qualification: though historical epistemology produced judgments, it did not aim at sanctions:

A judgment in this case is neither a purge nor an execution. The historiography of science is no backward-oriented history of progress [i.e., no Whig history], not a representation of episodes that, from the vanishing point of today’s truth, are obsolete. Rather it wants to investigate and to make intelligible to what extent notions, attitudes or methods which are nowadays obsolete were themselves innovations at their time and to what extent, as a consequence, the obsolete past remains the past of an activity which still deserves to be called scientific. It should not only become intelligible why something had been dismantled, but also how it was first constructed. (Canguilhem 1994a, p. 14; my translation).^17^

Like the laboratory metaphor, the metaphor of the tribunal conceived of the history of science as a field to retrieve data from, but the relation between data and theorizing had changed. Whereas the laboratory used history to test philosophical ideas about science, the tribunal assessed the past of scientific ideas in order to identify their errors.

To Bachelard, most of the history of science was about errors. In order to understand the current state of science, which in his view was a stage in a continuous movement of scientific progress, one needed to reflect on the histories of both the obsolete and the confirmed. Episodes or ideas that were obsolete from the present perspective might have destroyed wrong convictions at the time of their introduction. Progress in knowledge is not simply the occupation and structuration of the previously unknown, but to a much larger extent the replacement of the previously known. The realization of truth is inhibited not simply by non-knowledge, but by wrong pre-knowledge (cf. Schmidt and Tietz 1980, p. 10). Thus, while Bachelard insisted that the history of science was a history of progress, it nonetheless appeared as a series of breaks, of ruptures with ideas previously deemed true. To him, any attempt to display science as a continuously and cumulatively growing field would lead into a blind alley, into non-science, non-philosophy. Nobody could seriously claim an intellectual linkage between alchemistic transformation and nuclear transformation (Bachelard 1974, p. 76). Cumulation was a narrative artifact; ruptures were the very essence of scientific progress.

Several factors inhibited the process of scientific discovery, and since they were responsible for the existence of wrong pre-knowledge, the task of philosophy-as-tribunal was to identify them. Unlike other authors, Bachelard was less interested in genuinely social or political factors biasing science and emphasized psychological and cultural effects. He searched for epistemological obstacles, effects of the psychological inertia of the cognitive mind. In Bachelard’s view, the human mind formed habits of thinking that remained unreflected for the most part of our lives. While functional in everyday life, if brought to science, these obstacles could divert the cognitive processes from reaching the truth. Therefore, Bachelard claimed that a psychoanalysis of the scientific mind was needed. One type of epistemological obstacles were caused by primary experience. When a human being first experiences a thing, this experience is placed “before and above that criticism which is necessarily an integral part of the scientific mind.” (Bachelard 2002, p. 33) Primary experience thus had the potential to counteract and hinder scientific thinking. Another potential source was universalization, i.e. the attempt to transfer observational sentences into general statements of the highest order (e.g., scientific laws). Bachelard claimed that the idea to establish a new science by claiming fundamental principles and take these as basis for universal systems of thought had repeatedly obfuscated the process of science (Bachelard 2002, p. 65).

A third important source of epistemological obstacles was mythic thinking. Myths effectively confined the range of conceivable approaches to a phenomenon and thus could obviate epistemic progress, both on the individual and the collective level; a process that Bachelard extensively discussed with regard to fire (Bachelard 1964).^18^ One example of a myth that Bachelard saw influential across all scientific disciplines was the myth of the digestion: the belief that the truth of a thing could only be found deep in its interior and that one had to internalize it in order to fully grasp it (Bachelard 2002, pp. 172–184). Other examples for myths turning out to be epistemological obstacles that Bachelard mentioned were: “a sexualised view of nature, an attraction for small and precious objects, the instinct to possess these objects and the consequent desire for them to be ‘real’, and a disposition […] for attributing a soul, or at least life, to any object or substance.” (Chimisso 2003, p. 303).

For Bachelard, it was thus paramount to break with these pre-scientific pieces of knowledge, a step he called epistemological rupture. This meant to draw a radical break between everyday and scientific insight. Every statement must be checked for implicit, non-confirmed assumptions. Intuition was not a way to attain true knowledge, as Henri Bergson and other contemporary philosophers claimed (cf. Bachelard 2000). Also, science was not simply a continuation of everyday thinking.

Bachelard used the notion of an epistemological obstacle also to define the distinction between a traditional history of science and epistemology. “A fact that a whole era has misunderstood remains a fact in historians’ eyes. For epistemologists however, it is an obstacle, a counter-thought.” (Bachelard 2002, p. 27) While historians of science could content themselves with documenting a wrong truth, the historical epistemologists had to identify the factors leading to its acceptance and to assess whether the very same factors still influenced current research and theorizing. This was the normative, or prescriptive part of historical epistemology. Based on an examination of science’s past, it had to judge which parts of current science still rested on errors introduced by factors like culture, desires, or myths.

The standard used for this evaluation was “the standpoint of reason”. This standpoint of reason, however, was not informed by a historicist reconstruction of what “reason” meant at the time of the forging of an idea. Rather, Bachelard claimed that the evaluation had to be informed by actual, “developed” reason, “for it is only now that we can really judge the errors of the mind’s past.” (Bachelard 2002, p. 27). While the traditional history of science by principle – or prima facie? – negated any attempt to evaluate the historic events it deals with, the task of the epistemologist is to evaluate scientific ideas (Canguilhem 1981).

In an attempt to evade traditional relativism, Bachelardian historical epistemology applied the normative criteria provided by current epistemology to discern obsolete from confirmed insights. For Bachelard, there was no doubt that science progressed: progress in science to him was “undisputable,” a fact “withdrawn from any discussion.”^19^ Taking a provocative teleological stance, Bachelard was convinced that a science had a destiny, and not just a chronology (cf. Canguilhem 1994b, p. 175). And it was precisely because of this destiny that a philosophy of science could emerge from a history of science.^20^

To sum up, the Bachelardian version of historical epistemology engenders a normative historiography of science that is not only concerned with a Rankean *wie es eigentlich gewesen*, but discerns between correct and false ideas and analyzes the interplay between these ideas with the aim to inform current scientific practice. Such normative historiography emphasizes the importance of epistemological obstacles, of habits of thinking that are of an origin external to science and hinder the scientific progress. To explore these obstacles would allow for developing an epistemology that would anticipate and counteract their effects by convincing scientists of the ultimate need for epistemological rupture with their pre-scientific thinking.

## The Mediated and Partial Reception of Bachelard in Sociology

Bachelard and Canguilhem influenced a series of important French scholars.^21^ The most important of these from the perspective of sociology certainly were Michel Foucault and Pierre Bourdieu, who participated in courses by the two seniors and both wrote theses under the supervision of Canguilhem (although Bourdieu didn’t complete his). In their work on the relations of knowledge and power, both Foucault and Bourdieu emphasized the context of discovery over the context of justification, to borrow Hans Reichenbach’s distinction (cf. Tiles 1984, pp. 5–9). Also, the core project of a normative historiography was followed, albeit not in the sense proclaimed by Bachelard. Michel Foucault’s own archeology of knowledge (esp. Foucault 2002a, b), while lauding the reflexive stance of Bachelard/Canguilhem towards epistemology, deliberately moved out of the territory of science to question the very phenomenon of science. From the perspective of Foucault, an analysis á la Bachelard appeared to be bound to a scientific rationality and was thus unable to reflect on it. What is of interest for Foucault is to find a position from which to reflect on the phenomenon of science, and this stance must be located outside the epistemic realm of science as we now know it, outside science’s *episteme*. As he put it in *The Order of Things,* his project was to explore.

what modalities of order have been recognized, posited, linked with space and time, in order to create the positive basis of knowledge as we find it employed in grammar and philology, in natural history and biology, in the study of wealth and political economy. Quite obviously, such an analysis does not belong to the history of ideas or of science: it is rather an inquiry whose aim is to rediscover on what basis knowledge and theory became possible; within what space of order knowledge was constituted; on the basis of what historical a priori, and in the element of what positivity, ideas could appear, sciences be established, experience be reflected in philosophies, rationalities be formed, only, perhaps, to dissolve and vanish soon afterwards. (Foucault 2002a, p. xxiii).

Obviously, Foucault’s project thus was different from Bachelard’s. While Bachelard was concerned with the link between the history and the philosophy of science, science as the dominant mode of ordering things is the object of Foucault’s analysis. Yet, Foucault’s critique of Bachelard’s project is itself based on two Bachelard-inspired assumptions: first, that there are epistemological ruptures between epochs; and second, that certain questions cannot be addressed from within the dominant *episteme*, which thus has the effect of an epistemological obstacle. Finally, Foucault advanced a project that, while not being normative in the sense developed by Bachelard, had nonetheless undeniable an evaluative, and more precisely: political, message. His project was one of enlightenment, as Bachelard’s had been one of scientific progress.

A comparable mission of reflective enlightenment characterizes the works of Pierre Bourdieu. Well-known is his formulation that “one cannot avoid having to objectify the objectifying subject.” (Bourdieu 1988, p. xii) The scientist, and thus the sociologist, is a creator of classifications, and must as such become the object of reflective sociological analysis. Only such analysis enables the sociologist to gain a fruitful distance to her or his usual world. As his writings on methodological issues, and especially the essays in *The Craft of Sociology* (Bourdieu et al. 1991) evince, this is the place where the notion of epistemic rupture entered Bourdieu’s thinking. Here, epistemic rupture describes the capacity of the sociologist to break with the pre-scientific knowledge she or he has as a member of society, and has thus a meaning quite similar to Bachelard’s. And while Bourdieu, to my knowledge, did not use the term, it is apparent that if not reflected, the familiar world of the sociologist functions as some sort of epistemological obstacle, inhibiting a truly sociological analysis. Such analysis is only possible when the sociologist’s own social status, available forms of capital, and social biography enter the analysis, thus relativizing the objectifying position from which the analysis is carried out. While presented mainly as a methodological argument, this relativizing position nonetheless has obvious political and emancipatory implications.

There is yet another similarity between Bachelard/Canguilhem and Bourdieu, and it is in the latter’s concept of historical anamnesis. Bourdieu used this term in *The Rules of Art* (Bourdieu 1996) with regard to traces of presentist thinking in the history of art, but he also referred to it in his more political writings on history and power, where he argued that since the past influences the present in partly unacknowledged, yet no less powerful ways, historical anamnesis meant to debunk the myths of traditional historiography, which has tended to serve the nation-state and its power structures. “The work of anamnesis of the historical unconscious is the major instrument for gaining mastery of history, and therefore of the present that is an extension of history.” (Bourdieu 2008, p. 267) It is difficult to assess how much this concept of historical anamnesis owes to Bachelard/Canguilhem, but it certainly shares the idea that writing the history of a phenomenon goes beyond a *wie es eigentlich gewesen*, and instead has a diagnostic and normative-critical function. Despite this closeness, a second view suggests a subtle difference. This difference has become most clear in a recent book by Goldberg (2017), who used this concept as the starting point for his work in the history of social thought. Rather than focusing on the ruptures in the history (of science) or separating the history of the obsolete from the history of the confirmed, Bourdieu’s approach is to investigate the continuities from the past to the present. Thus, in line with Bourdieu’s understanding, Goldberg claims to “treat the present as an extension of history, albeit a history that is often forgotten and which thus exercises a hidden influence on the present.” And with reference to the Historicist Controversy, Goldberg continues: “This continuity between past and present is what allows one to apply the instruments of sociological analysis, which are a product of history, to the history of sociology itself.” (Goldberg 2017, p. 10).

Mediated mainly by Foucault and Bourdieu, some of Bachelard’s ideas are thus still discussed in sociology. This, however, does not apply to his program of historical epistemology in its entirety, and the ensuing section discusses three aspects of Bachelard’s program that have not been taken up. All three point to difficulties that proclaiming a historical epistemology of sociology implies.

## A Historical Epistemology of Sociology? Problems over Problems

As Fig. 1 shows, historical epistemology combines two movements of thought – the historization of epistemological elements, and the epistemologization of history. This follows many standard accounts of the field (e.g., Rheinberger 2010b) and is shared also by those who recently called for a historical epistemology of the social sciences and humanities. However, in historical epistemology, these two movements of thought are intrinsically and inseparably linked. Clearly, one can do HoS studies that follow just one movement, and the discussion above presented ample evidence showing the fruitfulness of such studies. But if one claims to do historical epistemology, then there is no way around acknowledging both movements and synthesizing them in a sensible way.

One way of synthesizing these two movements has been Bachelard’s open call for a normative history, and more importantly, for a normative history that is deliberately presentist. While, as I have argued, the approaches of Foucault and Bourdieu were evaluative, political, or emancipatory in their own ways, the tribunal function has not seen much support in contemporary HoS as well as in the history of science more generally, and it appears difficult to develop a convincing agenda for a prescriptive approach to the history of sociology. To be sure, there are studies that reflect on current practices or suggest rethinking basic assumptions in the discipline, as the remarks above showed. Yet, they rarely end with prescribing modifications to sociology’s current epistemology.

Further, to follow Bachelard’s program in sociology would be to seek for epistemological ruptures. While the notion of epistemological ruptures might be acceptable to the historian of sociology, the proposal that ruptures result in scientific progress enters a difficult terrain. Clearly, the idea of progress is operative on the level of individual scientific work in sociology. We need it to justify our doing to our readers and to ourselves (cf. Mozetič 2015). However, to determine progress in sociology on the level of the collective is unrealistic. Sociology’s objects are continuously changing, and the discipline thus continuously produces new schemes of interpretation. It is hard to assess whether these new schemes are novel, let alone whether they indeed are a progress in a true sense of the word. While this might become clearer in historical hindsight, the continuous transformation of the social world also distances us at a rapid pace from worlds of the past. Thus, it is difficult both to understand which other ideas had be overcome by the idea under scrutiny, and since they are themselves part of the cultural transformation, it is also difficult to fully grasp the psychological or psychoanalytical forces influencing past sociologists. Also, in many cases, what could be observed in the history of the social sciences is not a simple rise-and-fall pattern, but rather a cycle pattern of rise-and-fall-and-rise-again.

Finally, if one was to decide on a label for the attempt to follow Bachelard’s historical epistemology in writing the history of sociology, one could ponder about calling it a historical psychology of the sociological mind. Yet, this label smacks of an old-fashioned line of thinking, and rightly so, because it counters several methodological principles now common HoS. One of these principles, alluded to in the previous quote from Goldberg, is that one has to write the history of sociology from a sociological standpoint. Bachelard’s attempt to find psychological, or even psychoanalytical causes for the sustained acceptance of wrong knowledge is at odds with this principle. Yet in order to do historical epistemology, a consideration of such psychological causes appears inevitable.

These three ideas – the normative and presentist understanding of history, the idea of scientific progress, and the search for psychological factors – render it difficult, and at the moment apparently impossible, for historians of sociology to follow the comprehensive program of historical epistemology. At the same time, they don’t have to; as long as they do not claim to do historical epistemology, they can follow whatever paths they want and use whatever parts of historical epistemology’s rich tradition that they seem fit. Perhaps, the writings of Bachelard have something to offer them, too. More complicated is the situation with regard to those sailing under the banner of historical epistemology. To convincingly claim that there is more behind their use of this label than just academic entrepreneurial positioning requires a much higher awareness of the past (and present) of historical epistemology.

## Conclusion

In this paper I showed that there is a great variety of answers to the question how the history of sociology can contribute to current debates in sociology. Some scholars argue that HoS plays an important role in (I) shaping and maintaining the identity of the discipline. Identity politics consists in a continuous interplay of construction and critique, with both movements fostering the binding force of the disciplinary identity. HoS is also an important resource for (II) teaching sociology, where the classics remain valuable sources for training the next generations. Further, HoS insights can and should be used (III) to inform current research and theorizing. It can do so either by maintaining a back catalogue of sociological knowledge production and by providing, upon request, examples and advice to sociological practitioners – a service I called the card box function (III.1). It can explore the historical contingencies informing the formation of ideas and concepts, thus historicizing elements of sociology’s theory, methodology, and epistemology (III.2). Or it can use the history of sociology as a rich resource of material to address problems related to the sociality of knowledge production (III.3.a), the structurality of cultural (re-) production (III.3.b), or the psychology of the scientific mind (III.3.c). Finally, HoS also (IV) reflects on the status of sociology and sociological knowledge as part of modern societies and as a cultural force. Where available, I have discussed examples to further corroborate these claims.

Of course, the proposed scheme primarily has an analytical value. Most contributions to the mentioned debates argue on several levels that the scheme discerns. Notwithstanding, I hold that the scheme is of value for two reasons. Firstly, by structuring an ongoing and at the same time scattered debate amongst sociologists, it offers a synthesis of previous positions that might form the basis for the productive continuation of the debate. Secondly, by offering a systematic overview, the scheme also suggests potential approaches to writing the history of sociology which are not yet fully appreciated or utilized. While recent contributions to the history of sociology literature have shown an increasing interest in the sociality of knowledge production, dissemination, and evaluation, other perspectives, among them those focusing on cultural, structural, psychological, philosophical, or even psychoanalytical aspects of sociological research and theorizing, were taken less frequently. Without denying the importance of continuing the exploration of the social embeddedness of sociological research, we can still hold that the more we are aware of potential alternatives, the more we can reflect on the strength and weaknesses of the approaches we prefer. The scheme thus is intended to increase the level of methodological debate and reflexivity in the field.^22^

This article put considerable emphasis on the program of a normative historiography of science as it was developed by Gaston Bachelard and Georges Canguilhem. This program attempts to identify epistemological obstacles by comparing the history of confirmed insights with the history of scientific errors. Once identified, these epistemological obstacles should feed into a reformulation of scientific elements – of concepts, of theories, of methodologies, of epistemologies. While the program formulated by Bachelard and Canguilhem has only been followed partially in HoS, and then mainly via the mediation of Michel Foucault and Pierre Bourdieu, there certainly is potential to do so. Using the history of the Delphi technique, I have once described epistemic hopes – the expectations towards capacity, productivity, efficiency, and impact of a scientific idea that guide the author(s) of this idea in its creation and development – as epistemological obstacles, because they blind them for the idea’s errors and inadequacies (cf. Dayé 2015). However, another brief and necessarily sketchy example from the history of sociology might be useful here. In *The City*, Robert E. Park, Ernest W. Burgess, and Roderick D. McKenzie published collected papers that described their research program in and on Chicago (Park et al. 1984 [1925]). The book’s second chapter, “The Growth of the City” by Burgess, introduced the famous circle model of the modern city which he used then to explain their theory of expansion: while the innermost circle (“The Loop”) was home to hobos only, the newly arriving city dwellers sought housing in the second circle, thereby driving out those who had been living there. These decided to move to one of the better areas in the third circle, where they caused a similar reaction.

One can criticize the use of Chicago as a model of *the* modern city – Boston’s Beacon Hill and Manhattan’s Upper West and East Sides are famous counter examples for housing areas for the well-to-do in the first (or second) circle. Yet, one can also question the choice of a circle to describe the modern city. That Chicago itself fills only one half of the circle, with Lake Michigan as the other, is just the first in a series of peculiarities of the author’s choice of a circle. Another oddity is that a few chapters later, a similar model of apparently concentric circles is introduced. In “Community Organization and Juvenile Deliquency,” Park listed the different spheres of an individual’s social environment: first, the body; second, the individual’s “clothing, tools, and property” (Park et al. 1984, p. 101); third, the family; fourth, the neighborhood, and so on. It is not made explicit that the underlying notion is circular, but the discussion makes it clear that this is the mental image Park had. Again, as for instance Norbert Elias (1978) has shown, a circle is a rather tendentious and unrealistic way to describe individual’s social environment. Our relations are primarily to people, and people in figurations, as Elias would say, and we don’t have to cross the circle of family to reach school. Thus, we have, in the same book, two instances of selecting the very same mental image for theorizing two different cases; yet in both cases, this mental image lacks the power to convince of its applicability.

A Bachelardian analysis would now question the meaning of the circle as a symbol, and it would locate the ultimate reasons for choosing the circle in its cultural connotations. And indeed, this is not a bad fit, if one is ready to follow the depth psychology of C. G. Jung and others. Circles (or spheres) symbolize wholeness, the completeness, integrity, and harmony of the self, as well as sometimes healing and the halo of God (cf. Jung 1964). This, of course, fits well with the social problems Park and his colleagues dealt with: the rising numbers in poverty, the decline of the family as a place for moral education, and juvenile delinquency. Thus, under the condition that one feels confident enough with psychoanalysis and depth psychology, one can make the claim that the unconscious motive for the choice of circles by the Chicago sociologists was the wish to improve – heal – the social ills they perceived in Chicago (and elsewhere).

It should be clear that these remarks amount to no more than conjectures, but they might nonetheless serve as an example how a psychology of the scientific mind (III.3.c) might work out in HoS. Again, HoS has the advantage *vis-à-vis* those claiming to do historical epistemology that it can remain eclectic the corpus of historical epistemology. Those advocating a historical epistemology of the social sciences and humanities, however, must approach this corpus in a more catholic manner.

Considering the whole range of capacities of HoS to inform contemporary debates in sociology, it appears reasonable to conceive of HoS as a crucial part of sociology. This claim is neither a form of strategic boundary demarcation vis-à-vis historians nor is it an attempt to secure the intellectual vitality of the history of sociology. On the contrary, historians have recently provided excellent contributions to the history of the social sciences. What is more, there is a vibrant meta-discourse within history on why and how the history of science should be written (for an overview, see Erickson 2010a; as well as the ensuing debate with contributions by Fara 2010; Fuller 2010; Rouse 2010; and a response by Erickson, 2010b). Thus, HoS itself is not endangered. If sociological associations decided to close the sections devoted to their own history; if sociological curricula continued to marginalize the history of sociology; and if major sociological journals continued to reject historical work for lack of fit into the methodological mainstream of the discipline, then the gravitational point of HoS would certainly move. Yet, the field would not disappear. However, as I hope to have shown, this move out of the discipline would be a loss to sociology.

## Footnotes

1 Unfortunately, the term “history of sociology” denotes both a scientific field (in an unspecific sense) and an object of study. Sociology has a history, and it is studied by members of the history of sociology community. I will use the acronym “HoS” to refer to the field only.

2 The ensuing summary of events is based on available materials as well as a personal communication with Alan Sica (via email, September 2017).

3 Matters were worsened by the judgment of a lawyer specialized in intellectual property rights, who argued that most of the materials (especially unpublished manuscripts and reviews) were still the property of their authors, and to make these materials accessible would require to secure the agreement of the authors or their heirs. Two memoranda document the debate and ultimately the decision of the ASA leaders: the “President’s Memorandum to Membership on Council Decision regarding Editorial Paper Records” (March 1, 2014) and the “Memorandum on the Disposition of Editorial Office Files From 1991 to 2009” (March 1, 2014), both available at http://www.asanet.org/asa-statements, accessed June 11, 2018.

4 The website used to collect these statements, saveourarchivalrecords.org, is currently inaccessible (July 7, 2018).

5 The outcome of these debates was mixed. While the History of Sociology section continues to exist, ASA management decided not to pay for the preservation of the journal records, but allowed the “Save Our Archival Records” initiative led by Charles Camic and Alan Sica to seek for “interested parties” willing to donate. While the fund-raising campaign succeeded in collecting the required amount of money, Camic and Sica were excluded from the further proceedings, the current status of which remains unclear.

6 The “Historicist Controversy” played out in contributions by Jones (1974, 1977, 1981, 1983a, b, 1985); Camic (1979, 1981); Seidman (1985); Turner (1985); Warner (1985). The debate in the Swiss Journal of Sociology consisted of papers by Hirschhorn (1997); Valade (1997); Camic (1997); Jones (1997); Turner (1998); Sica (1998); Kaesler (1999).

7 Among the book-length contributions to this literature by historians over the past ten years, one might mention Cohen-Cole (2014); Erickson (2015); Erickson et al. (2013); Haney (2008); Heyck (2015); Igo (2007); Isaac (2012); Mandler (2013); Rohde (2013); Rindzevičiūtė (2016); Solovey (2013); Solovey and Cravens (2012); Thomas (2015).

8 Apart from the Max-Planck-Institut für Wissenschaftsgeschichte in Berlin (http://www.mpiwg-berlin.mpg.de/, accessed June 19, 2018), where Daston and Renn are leading figures, academic units concerned with the historical epistemology of the social sciences and humanities can be found at the Institute for German Literature of the Humboldt-Universität zu Berlin (http://fheh.org/, accessed June 19, 2018) and at the Forschungszentrum für Historische Geisteswissenschaften of the Johann-Wolfgang-Goethe-Universität in Frankfurt am Main (http://fzhg.org/forschungsfelder/ff1-historische-epistemologie/, accessed June 19, 2018).

9 It is also questionable whether their programs are really superior to the sociology of scientific knowledge and other sociological programs in the history of science (cf. Kinzel 2012).

10 I borrow the term “strategic research materials” from Robert K. Merton (1987).

11 Together with John Holmwood, Stephen Turner is in the process of editing a series of national histories published by Palgrave MacMillan, called “Sociology Transformed” (http://www.palgrave.com/in/series/14477, last visited August 31, 2017). At the moment, there exist volumes on Australia (Harley and Wickham 2014); Austria (Fleck 2016); Belgium (Vanderstraeten and Loucks 2018); China (Chen 2018); the Czech Republic (Skovasja and Balon 2017); Denmark (Kropp 2015); France (Masson and Schrecker 2016); Ireland (Fanning and Hess 2015); Israel (Ram 2018); Italy (Cossu and Bortolini 2017); New Zealand (Crothers 2018); Poland (Bucholc 2016); Portugal (da Silva 2016); Russia (Titarenko and Zdravomyslova 2017); South Africa (Sooryamoorthy 2016); Sweden (Larsson and Magdalenić 2015); and the United States (Turner 2014). Further books are in preparation. Several of the books in this series share the resource-oriented perspective developed in *The Impossible Science* (Turner and Turner 1990).

12 See, for instance, the INTERCO-SSH project (http://interco-ssh.eu/en/, accessed June 19, 2018).

13 The history of the label is itself complicated. Bachelard appears to have never used it (Canguilhem did), but a thesis written by Dominique Lecourt under the guidance of Canguilhem and later published both in French and English explored “Gaston Bachelard’s Historical Epistemology” (Lecourt 1975). An instructive overview over the history of the label has been offered by Gingras (2010).

14 The term “normative” might be mistakable, but it is a term used by Canguilhem describing Bachelard’s approach, and I therefore decided to keep it. There might also be some translation issues involved, and in English, the term “evaluative” might describe the similarity between Bachelard/Canguilhem and Foucault/Bourdieu more accurate.

15 Here, I fully agree with Cristina Chimisso’s remarks: “Canguilhem advocated the normative approach to history of science in several articles and talks. When he wrote his theoretical remarks on history of science and epistemology, the normative turn in my view had been fully realized. The epistemological approach had disentangled itself from the ideal of total history and had chosen normativity over a relativistic and philological approach. The goal of combining the historical craft with philosophical aims had been abandoned, and the tension between these two poles of history of science had been resolved. It had been resolved thanks to the success of the epistemological approach in the hands of Gaston Bachelard, closely followed by Canguilhem. In his theoretical essays, Canguilhem indeed relied on the ideas and authority of his predecessor in the Sorbonne chair of history and philosophy of the sciences. Indeed, Canguilhem made important reflections on this type of history in articles on Bachelard.” (Chimisso 2003, p. 310)

16 In contrast to all other branches of philosophy who had to construct systems, philosophy of science thus could be understood as “la seule *philosophie ouverte*”, the only open philosophy (Bachelard 1966, p. 7).

17 „Un jugement, en cette matière, n’est. pas une purge, ni uns exécution. L’histoire des sciences ce n’est. pas le progrès des sciences renversé, c’est-à-dire la mise en perspective d’étapes dépassées dont la vérité d’aujourd’hui serait le point de fuite. Elle est. un effort pour rechercher et faire comprendre dans quelle mesure des notions ou des attitudes ou des méthodes dépassées ont été, à leur époque, un dépassement et par conséquent en quoi le passé dépassé reste le passé d’une activité à laquelle il faut conserver le nom de scientifique. Comprendre ce que fut l’instruction du moment est. aussi important qu’exposer les raisons de la destruction par la suite.” (Canguilhem 1994a, p. 14)

18 In *The Psychoanalysis of Fire*, Bachelard (1964) discussed a series of psychoanalytic and psychological complexes that influence cognition, among them the Prometheus complex, the intrinsic wish to know more than our fathers and teachers.

19 “On peut discuter sans fin sur le progrès moral, sur le progrès social, sur le progrès poétique, sur le progrès du bonheur; il y a cependant un progrès qui échappe à toute discussion, c’est. le progrès scientifique dès qu’on le juge dans la hiérarchie des connaissances, en son aspect spécifiquement intellectuel.” (Bachelard 1966, p. 21)

20 “Une science a son destin et non seulement une chronologie. De l’histoire de la science, philosophiquement questionnée, c’est-à-dire quant à la formation, à la réformation et à la formalisation des concept, surgit une philosophie de la science.” (Canguilhem 1994b, p. 175)

21 Although similarities exist, it should be clear that compared to, for instance, Alexandre Koyré’s (1957) concept of a scientific revolution or the change of paradigms that Thomas Kuhn (1970) saw to occur in such revolutions, Bachelard’s notion of epistemological rupture referred to a different kind of discontinuity. Both Koyré and Kuhn were concerned with science as a collective and did not include the scientific individual in their analyses. Also, while epistemological ruptures for Bachelard were radical on the individual level, and per force had to be radical in order to allow for scientific progress, they were always partial on the collective level and did not embrace the totality of a (scientific) weltanschauung, as in Koyré’s *From the Closed World to the Infinite Universe* (Koyré 1957). Finally, in Bachelard’s writings, we find nothing comparable to Kuhn’s notion of incommensurability, a term by which he denoted a break in the development of a science which rendered impossible any form of epistemic continuity. The historical and theoretical relations between Kuhn and the French epistemologists are discussed in more detail in Brenner (2006); Simons (2017).

22 A comparable, but less comprehensive scheme has been proposed by German sociologist Frank Welz (2010). Welz discerns three types of historian of sociology, which he dubs identity engineer, collector of ideas, and trace-tracker. These types fit into the scheme power in this article (categories I.1, I.2, and III.2, respectively). However, Welz has been criticized for using Robert K. Merton as example of a positivist historian of sociology (cf. Dayé 2012; Welz 2012).
